# Gab1 is essential for membrane translocation, activity and integrity of mTORCs after EGF stimulation in urothelial cell carcinoma

**DOI:** 10.18632/oncotarget.2756

**Published:** 2015-01-20

**Authors:** Chi-Hao Chang, Po-Chao Chan, Jian-Ri Li, Chun-Jung Chen, Jeng-Jer Shieh, Yun-Ching Fu, Hong-Chen Chen, Ming-Ju Wu

**Affiliations:** ^1^ Institute of Clinical Medicine, National Yang Ming University, Taipei, Taiwan; ^2^ Department of Life Sciences, National Chung Hsing University, Taichung, Taiwan; ^3^ Department of Surgery, Division of Urology, Taichung Veterans General Hospital, Taichung, Taiwan; ^4^ Department of Education and Research, Taichung Veterans General Hospital, Taichung, Taiwan; ^5^ Institute of Biomedical Science, National Chung Hsing University, Taichung, Taiwan; ^6^ Department of Pediatrics, Taichung Veterans General Hospital, Taichung, Taiwan; ^7^ Department of Medicine, Division of Nephrology, Taichung Veterans General Hospital, Taichung, Taiwan; ^8^ School of Medicine, Chung Shan Medical University, Taichung, Taiwan; ^9^ Graduate Institute of Clinical Medical Science, School of Medicine, China Medical University, Taichung, Taiwan

**Keywords:** mTORCs, Gab1, EGF, urothelial carcinoma

## Abstract

Urothelial carcinoma is the most common type of malignancy in long-term dialysis patients and kidney transplant recipients in Taiwan. mTORCs (mammalian target of rapamycin complexes) and EGF are important in urothelial carcinoma. To identify the regulation of mTORCs upon EGF stimulation is necessary. mTOR integrates signals from growth factors via mTOR Complex 1 (mTORC1) and mTOR Complex 2 (mTORC2). The mechanism of mTORC1 action has been widely studied; however, the regulation of mTORC2 has not been well studied. Here, we demonstrate that Gab1 is an important upstream regulator in EGF-mediated activation of mTORCs. In our study, we confirm that mTORCs translocate from the cytoplasm to the plasma membrane via the PH domain of Gab1 upon EGF stimulation. Moreover, Gab1 associates with mTORCs. This association stabilizes the integrity of mTORCs and induces mTORC activity. Compared to normal bladder tissue, the expression of Gab1 and activity of mTORCs are elevated in urothelial carcinoma. Collectively, our results suggest that Gab1 is an essential regulator of the EGF-mediated mTORC pathways and may potentially be used as a biomarker for urothelial carcinoma to predict diagnosis and drug response.

## INTRODUCTION

Urothelial carcinoma is the most common type of malignancy in long-term dialysis patients and kidney transplant recipients in Taiwan [1]. Recently, we demonstrate that rapamycin, the mammalian target of rapamycin (mTOR) inhibitor, exhibits potent anti-tumor properties against BBN-induced urothelial carcinoma [2]. Accordingly, mTOR is important in urothelial carcinoma and identification of the mechanism of mTOR regulation in urothelial carcinoma is necessary.

The mammalian target of rapamycin (mTOR) is a serine/threonine protein kinase that plays a critical role in the downstream networks of many growth factor receptors [3, 4]. Two major regulators of the mTOR complex (mTORC) signaling pathways are mTORC1 and mTORC2. mTORC1 is rapamycin-sensitive and contains mTOR, raptor and mLST8, while mTORC2 contains mTOR, rictor, mLST8 and sin1 [5]. mTORC1 promotes mRNA translation via p70S6K to regulate protein synthesis and cell metabolism [6]. mTORC2 plays an important role in phosphorylation and subsequent activation of AKT to promote cell survival and proliferation [7–9].

It is known that mTORC1 is regulated by activation of PI3K, AKT, Rheb and inhibition of tuberous sclerosis protein 1/2 (TSC1-TSC2); however, the regulatory mechanisms of mTORC2 have not been completely elucidated [10]. PI3K plays a crucial role in mTORC2 activation. In an *in vitro* mTORC2 kinase assay, mTORC2 was directly activated by PI3K-phosphatidylinositol-3, 4, 5-trisphosphate (PIP3) in HEK293T cells [11]. Ribosomes induced by insulin associate with mTORC2 and lead to activation of mTORC2 [12]. Recently, we demonstrated that mTORC2-dependent AKT activation in a rat model of urothelial carcinoma is a consequence of mTORC1 inhibition [2]. mTORC1 activates p70S6K to phosphorylate rictor at Thr1135, leading to a reduction in mTORC2 activity [13]. Indeed, mTORC1 also plays an important role in mTORC2 regulation. ER stress activates GSK3β to phosphorylate rictor at Ser1235 and subsequently reduces mTORC2 activity [14]. In addition to phosphorylation of rictor, phosphorylation of sin1 attenuates mTORC2 activity. p70S6K and AKT induce phosphorylation of sin1 on Thr86 and Thr398 to dissociate sin1 from the mTORC2 complex [15]. According to these studies, the phosphorylation of rictor and sin1 appears to inhibit the activity of mTORC2; however, the role of the phosphorylation sites on rictor and sin1 remains unclear. The small GTPase Rac1 regulates both mTORC1 and mTORC2 in response to growth factor stimulation. Rac1 binds directly to mTOR independently of the GTP-bound state of Rac1 and mediates membrane localization of mTORCs [16]. The TSC1-TSC2 complex, a negative regulator of mTORC1, is required for activation of mTORC2. This complex associates with mTORC2 but not mTORC1, inhibiting mTORC1 and activating mTORC2 [17].

Previous reports have shown that epidermal growth factor (EGF) induce the activation of mTORC2 [18]. Downstream of the EGF receptor (EGFR), Grb2-associated binder 1 (Gab1) is a major adaptor protein for the EGF-mediated signaling pathway [19, 20]. Evidence suggests that Gab1 may potentially be an important player in the regulation of mTORC2 after EGF stimulation [18]. Gab1 belongs to a family of docking proteins that includes Gab2 and Gab3 [21], and it contains an NH2-terminal pleckstrin homology domain, a Met-binding domain, 3 proline-rich sequences and 16 potential tyrosine phosphorylation sites that represent Src-homology (SH) 2 domain-binding motifs [19, 21]. The PH domain is a common structural element in Gab family members that can recognize and bind to membrane proteins [22]. Moreover, the PH domain of Gab1 binds to PIP3, and is responsible for its membrane localization. It has been reported that the PH domain of Gab1 appears to be required for Gab1 protein function [23–25]. A PH domain deletion mutant of Gab1 is defective in EGFR and c-MET signaling [24].

A high percentage of EGFR-positive cases (up to 50% of cases) were observed in high-grade urothelial carcinoma [26]. These data suggest that EGFR plays a crucial role in urothelial carcinoma. However, the mechanism of Gab1 action in EGF-mediated activation of mTORC2 is unclear. In the present study, the critical role for Gab1 in EGF-mediated activation of mTORC pathways and the importance of Gab1 and mTORCs in urothelial carcinoma were investigated.

## RESULTS

### Elevated expression of Gab1 is associated with increased activation of mTORCs in urothelial cell carcinoma

**Figure 1:**
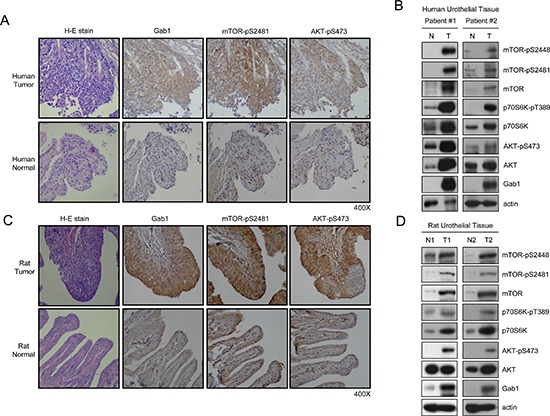
Elevated expression of Gab1 is associated with increased activation of mTORCs in urothelial carcinoma **(A, B)** Immunohistochemical staining was performed on paraffin-embedded human urothelial carcinoma tissues and normal urothelial tissues. The sample pictures of immunohistochemical staining of Gab1, mTOR-pS2481 and AKT-pS473 are shown (400X). The lysates from human urothelial carcinoma tissues and normal urothelial tissues were immunoblotted to detect mTOR-pS2448, mTOR-pS2481, p70S6K-pT389, AKT-pS473 and Gab1. N, Normal. T, Tumor. **(C, D)** Paraffin-embedded tissues from both normal control rats and urothelial carcinoma rats induced by 0.05% N-butyl-N-(4-hydroxybutyl) nitrosamine (BBN) were immunohistochemically stained for Gab1, mTOR-pS2481 and AKT-pS473 (400X). The lysates from normal control rats and rats with urothelial carcinoma were immunoblotted to detect mTOR-pS2448, mTOR-pS2481, p70S6K-pT389, AKT-pS473 and Gab1. N, Normal. T, Tumor.

Urothelial carcinoma is the most common type of malignancy in long-term dialysis patients and kidney transplant recipients in Taiwan [1]. Recently, we reported significant activation of mTORCs in urothelial carcinoma [2]. In this study, we used urothelial carcinoma cells to demonstrate that Gab1 was important for mTORC activation. Here, we examined the expression of Gab1 in many cells lines from different grades of urothelial carcinoma. Indeed, the expression level of Gab1 was found to be weaker in normal urothelial cell lines (E6, normal) and low-grade urothelial carcinoma cell lines (RT4, Grade I; TSGH8301, Grade II) and higher in high-grade urothelial carcinoma cell lines (J82, T24, Grade III) (Figure S1A). We then examined human bladder tissues with urothelial carcinoma (Figure 1A and 1B) and rat bladder tissues with urothelial carcinoma induced by 0.05% N-butyl- N-(4-hydroxybutyl) nitrosamine (BBN) (Figure 1C and 1D). As expected, immunohistochemical staining showed significantly increased expression of Gab1, phosphorylation of mTOR at Ser2481 and phosphorylation of AKT at Ser473 in both human and rat bladder tumor tissues but not in normal bladder tissues. Western blot analysis also confirmed these findings. Not only the mTORC2 activity, the mTORC1 activity (phosphorylation of mTOR at Ser2448 and phosphorylation of p70S6K at Thr389) also significantly increased in both human and rat bladder tissues. Furthermore, knockdown of Gab1 inhibited T24 cells colony formation (Figure S1B) and migration (Figure S1C). Taken together, our results indicate that both Gab1 and mTORCs are important in urothelial carcinoma.

### Gab1 is important for plasma membrane translocation of mTORCs after EGF stimulation

**Figure 2:**
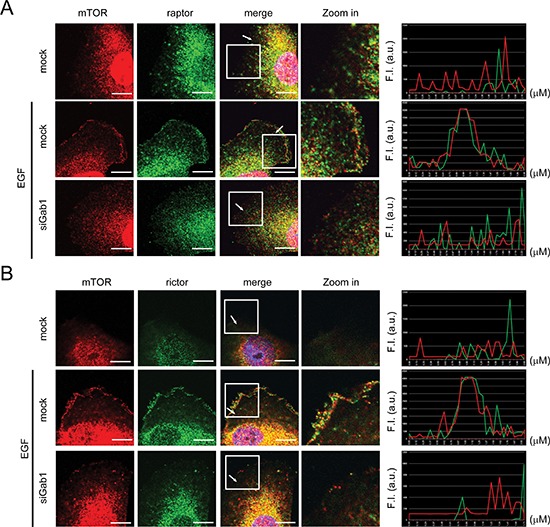
Knockdown of Gab1 reduces plasma membrane translocation of mTORCs after EGF stimulation **(A)** T24 cells transfected with Gab1 siRNA were serum-starved and treated with 100 ng/ml EGF (10 min). To detect the localization of mTORC1 in T24 cells, immunofluorescence staining was performed. Staining of mTOR (red), raptor (green) and DAPI (blue) are shown. The right panel shows the relative fluorescence intensity (F.I.) at the lines that were scanned by confocal microscopy. a.u., arbitrary unit. **(B)** The experiments were performed in a manner similar to that described in **A**. To detect the location of mTORC2 in T24 cells, immunofluorescence staining was performed. Staining of mTOR (red), rictor (green) and DAPI (blue) are shown. The knockdown efficiency of Gab1 by Gab1 siRNA was shown in Figure S2. m, mock. Scale bar, 10 μm. All experiments, *n* = 3.

**Figure 3:**
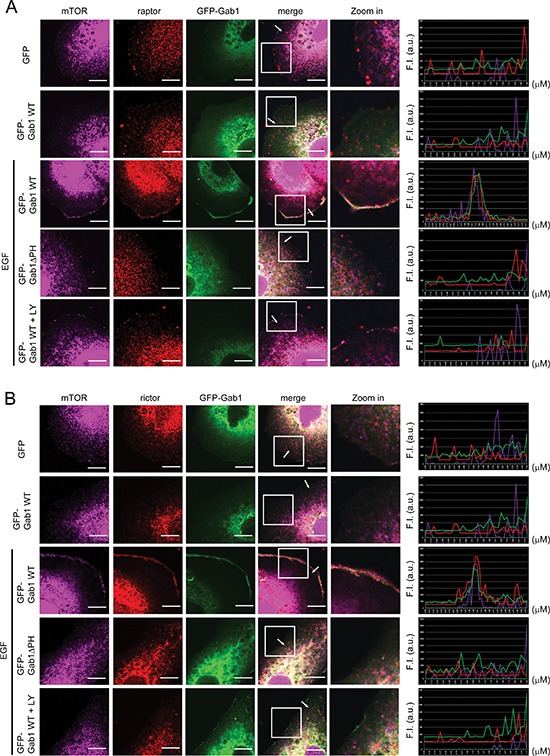
Overexpression of Gab1 promotes plasma membrane of mTORCs after EGF stimulation **(A)** E6 cells transfected with GFP-Gab1wt and GFP-Gab1ΔPH were serum-starved and treated with 100 ng/ml EGF (10 min) and 20 μM LY294002 (60 min). To detect the localization of mTORC1 and Gab1 in E6 cells, immunofluorescence staining was performed. Staining of mTOR (purple), raptor (red), Gab1 (green) and DAPI (blue) are shown. The right panel shows the relative fluorescence intensity (F.I.) at the lines that were scanned by confocal microscopy. a.u., arbitrary unit. **(B)** The experiments were performed in a manner similar to that described in **A**. To detect the localization of mTORC2 and Gab1 in E6 cells, immunofluorescence staining was performed. Staining of mTOR (purple), rictor (red), Gab1 (green) and DAPI (blue) are shown. m, mock. LY, LY294002. Scale bar, 10 μm. All experiments, *n* = 3.

In the EGFR signaling pathway, Gab1 is a major downstream docking protein triggered by EGF [19, 20]. After EGF stimulation, Gab1 translocates to the plasma membrane and activates downstream signaling pathways [23]. Moreover, mTORC2 is directly activated by PIP3 in HEK293T cells [11]. In the current study, we used Gab1 siRNA to knock down endogenous Gab1 in T24 cells, and we found that mTOR and raptor could not translocate to the plasma membrane after EGF stimulation (Figure 2A). Next, we overexpressed exogenous Gab1 in E6 cells, which have lower levels of Gab1 expression compared to T24 cells. Moreover, ectopic expression of green fluorescent protein-fused Gab1 (GFP-Gab1) in E6 cells facilitated the membrane translocation of mTOR and raptor after EGF stimulation (Figure 3A). In contrast, expression of a Gab1 mutant lacking the PH domain (Gab1ΔPH) or treatment with the PI3K inhibitor LY294002 suppressed membrane translocation of both proteins after EGF stimulation (Figure 3A). Interestingly, mTORC2 showed the same result upon EGF stimulation (Figure 2B and 3B). These findings provide evidence demonstrating that Gab1 regulates mTORCs by translocating to the plasma membrane after EGF stimulation.

### Gab1 is essential for activation of mTORC signaling after EGF stimulation

**Figure 4:**
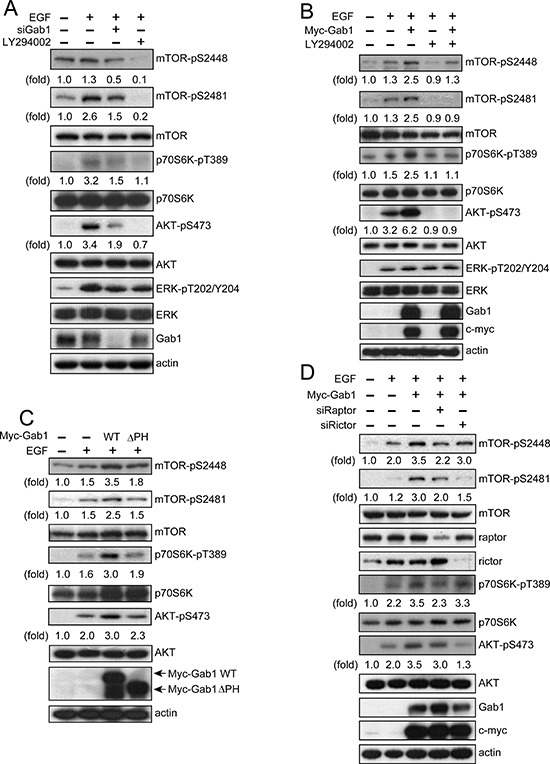
Gab1 is important for the activation of mTORC signaling after EGF stimulation **(A)** T24 cells were transfected with Gab1 siRNA, treated with LY294002 (20 μM) for 60 min, stimulated with 100 ng/ml EGF for 10 min and then immunoblotted to detect mTOR-pS2448, mTOR-pS2481, p70S6K-pT389, AKT-pS473, ERK-pT202/Y204 and Gab1. **(B)** E6 cells transfected with Myc-Gab1wt were serum-starved, and the treatment was performed in a similar manner to that described in **A**. Immunoblotting was performed to detect mTOR-pS2448, mTOR-pS2481, p70S6K-pT389, AKT-pS473, ERK-pT202/Y204, Gab1 and myc. **(C)** E6 cells were transfected with Myc-Gab1wt and Myc-Gab1ΔPH, treated with 100 ng/ml EGF for 10 min and then immunoblotted to detect mTOR-pS2448, mTOR-pS2481, p70S6K-pT389, AKT-pS473 and Gab1. **(D)** T24 cells were transfected with raptor siRNA, rictor siRNA and Myc-Gab1wt, treated with 100 ng/ml EGF for 10 min and then immunoblotted to detect mTOR-pS2448, mTOR-pS2481, raptor, rictor, p70S6K-pT389, AKT-pS473, Gab1 and myc. For all experiments, *n* = 3.

We established that Gab1 is important for plasma membrane translocation of mTORCs after EGF stimulation. Next, we examined the role of Gab1 in the regulation of mTORC activity. First, we knocked down endogenous Gab1 in T24 cells, and we found that the activities of mTORC1 and mTORC2 were inhibited after EGF stimulation. Furthermore, a PI3K inhibitor (LY294002) inhibited the activity of the mTORCs after EGF stimulation (Figure 4A). Next, we overexpressed exogenous Gab1 in E6 cells, which have lower levels of Gab1 expression compared to T24 cells. We found that overexpression of Gab1 increased the activity of mTORC1 and mTORC2 after EGF stimulation, and LY294002 also inhibited mTORC activities (Figure 4B). As shown in Figure 4A and 4B, inhibition of PI3K and Gab1 decreased mTORC activity after EGF stimulation. We then overexpressed the Gab1ΔPH mutant in E6 cells, and we found that overexpression of Gab1ΔPH decreased mTORC1 and mTORC2 activity after EGF stimulation (Figure 4C). We also knocked down raptor and rictor in T24 cells. mTORC1 activity was inhibited by raptor knockdown, and mTORC2 activity was inhibited by rictor knockdown (Figure 4D). These data provide direct evidence demonstrating that Gab1 is essential for the activation of mTORC1 and mTORC2 after EGF stimulation.

### Gab1 associates with mTORCs

**Figure 5:**
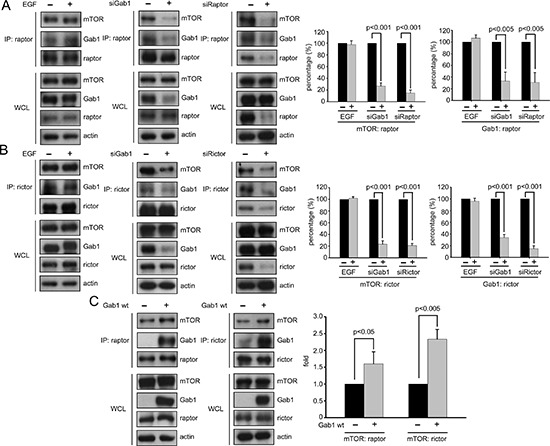
Gab1 forms complexes with mTORCs **(A)** Some T24 cells were serum-starved and then treated with 100 ng/ml EGF for 10 min, and other T24 cells were transfected with Gab1 and raptor siRNA and serum starved. Next, immunoprecipitation of raptor proteins with anti-raptor antibody was performed, and the supernatants from the immunoprecipitations were immunoblotted to detect mTOR, raptor and Gab1. The whole cell lysates (WCLs) were analyzed to detect the same proteins via immunoprecipitation. Quantification of immunoblotting was performed (mean ± SD; *p* < 0.005). The negative control was shown in Figure S3A. **(B)** Some T24 cells were serum-starved and then treated with 100 ng/ml EGF for 10 min, and other T24 cells were transfected with Gab1 and rictor siRNA and serum starved. Next, immunoprecipitation of rictor proteins with anti-rictor antibody was performed, and the supernatants from immunoprecipitation were immunoblotted to detect mTOR, rictor and Gab1. WCLs were analyzed to detect the same proteins via immunoprecipitation. Quantification of immunoblotting was performed (mean ± SD; *p* < 0.001). The negative control was shown in Figure S3B. **(C)** The E6 cells transfected with Myc-Gab1wt were serum starved, and immunoprecipitation of raptor and rictor proteins with raptor and rictor antibodies was performed. The supernatants from the immunoprecipitation were immunoblotted to detect mTOR, raptor, rictor and Gab1. The WCLs were analyzed to detect the same proteins via immunoprecipitation. The right panel shows quantification of immunoblotting (mean ± SD; *p* < 0.05). The negative control were shown in Figure S3C and S3D. The All experiments, *n* = 3.

It has been previously shown that Gab1 translocates to the plasma membrane after EGF stimulation [23], and we also showed that plasma membrane translocation of mTORCs occurs via Gab1 after EGF stimulation (Figure 2 and 3). We next questioned how Gab1 regulates the plasma membrane translocation of mTORCs. As shown in recent studies, many proteins, such as RalB and Rac1, may associate with mTORCs and regulate the plasma membrane translocation of mTORCs [16, 27]. To identify a potential association between Gab1 and mTORCs, we used co-immunoprecipitation. First, we found that Gab1 associated with mTORCs after EGF stimulation (Figure 5A and 5B). Interestingly, Gab1 also associated with mTORCs in the absence EGF stimulation. These data suggest that Gab1 had previously associated with mTORCs in cells. Then, we also knocked down Gab1 in T24 cells. We found that knockdown of Gab1 decreased the integrity of mTORCs in T24 cells (Figure 5A and 5B). Knockdown of raptor or rictor decreased the integrity of mTORCs and also decreased the association of Gab1 with mTORCs (Figure 3A and 3B). According to these data, Gab1 not only regulates the plasma membrane translocation of mTORCs but also stabilizes the integrity of mTORCs. Furthermore, we overexpressed Gab1 in E6 cells, and we found that Gab1 overexpression increased the amount of mTOR complexes in cells (Figure 5C). Thus, we demonstrated that Gab1 associated with mTORCs and stabilized their integrity. Furthermore, these functions were EGF-independent.

## DISCUSSION

Urothelial carcinoma is the most common type of malignancy in long-term dialysis patients and kidney transplant recipients in Taiwan [1]. mTORCs pathway is important in urothelial carcinoma [28] and it already known that EGF triggers mTORCs activation [18]. A high percentage of EGFR-positive cases (up to 50% of cases) were observed in high-grade urothelial carcinoma [26]. These data suggest that EGFR plays a crucial role in urothelial carcinoma and Gab1 is a major downstream protein in EGFR signal transduction pathway. Therefore, Gab1 may play an important role in regulating EGF-mediated mTORC activity in urothelial carcinoma. Not only urothelial carcinoma, it has been reported that mTORCs are activated in many different carcinomas, such as renal cell carcinoma, melanoma and prostate tumors [29, 30]. mTORC1 promotes HIF-1α and VEGF protein and mRNA expression to induce angiogenesis in renal cell carcinoma [31]. Through the activation of AKT and SGK, mTORC2 may directly drive tumorigenesis. AKT promotes proliferation, survival and nutrient uptake in tumor cells [32]. Furthermore, rictor promotes the assembly and activity of mTORC2, which is required for the growth of various tumor cell lines and for the growth of prostate tumors in PTEN-deficient mice [30]. In this study, we indicate the importance of regulation of mTORCs via Gab1 after EGF stimulation.

Several studies have indicated that growth factor stimulation is involved in the regulation of mTORC2. Currently, it is thought that mTORC2 is activated via the PI3K pathway. Zinzalla et al. (2011) revealed that in the PI3K pathway, the association with ribosomes generates signals that are transduced to the mTORC2 signaling pathway [12]. In fact, mTORC2 is also regulated by mTORC1. Julien et al. (2009) and Liu et al. (2013) revealed that mTORC1-activated p70S6K phosphorylated rictor at Thr1135 and phosphorylated sin1 at Thr86 and Thr398 to inhibit mTORC2 activity [13, 15]. Our previous report also showed similar findings, in which rapamycin inhibited mTORC1 and induced phosphorylation of AKT at Ser473 in urothelial carcinoma [2]. In addition to mTORC1, Chen et al. (2011) also revealed that ER stress inhibits mTORC2 through GSK-3-mediated phosphorylation of rictor at Ser1235 [14].

However, it has been reported that mTORC2 is directly activated by PIP3 in HEK293T cells [11]. Thus far, there is no evidence to indicate that signaling extends beyond PIP3 in the activation of mTORC2. Cao et al. (2009) revealed that EGF exerts its effects via Gab1 by means of an unidentified mechanism to regulate mTORC2 activity [18]. In fact, Gab1 is a major protein that associates with PIP3 after EGF stimulation [19]. In this study, we provide evidence to support the hypothesis that Gab1 associates with mTORC2 and regulates mTORC2 membrane translocation. When PI3K is inhibited, mTORC2 does not translocate to the plasma membrane after EGF stimulation. Moreover, Gab1 also associates with mTORC1 and regulates mTORC1 membrane translocation. We also revealed that PI3K activates mTORCs via the PH domain of Gab1, which binds to PIP3.

Interestingly, Gab1 not only associates with mTORCs and regulates their plasma membrane translocation but also stabilizes the integrity of mTORCs. Only the components of mTORCs (raptor, rictor and sin1) have been known to regulate the integrity of mTORCs [5]. In fact, there are two endogenous inhibitors of mTORCs, DEPTOR and XPLN. Depletion of DEPTOR activates mTORCs, and mTOR negatively regulates the protein and mRNA expression of DEPTOR [33]. XPLN is a guanine nucleotide exchange factor (GEF) for Rho GTPases and is a partner of mTORC2 but not mTORC1. Knockdown of XPLN enhances the activity of mTORC2 [34]. However, DEPTOR and XPLN are not similar to raptor, rictor and sin1. DEPTOR and XPLN interact with mTORCs and inhibit their activity. In this study, we found that Gab1 associates with mTORCs and regulates their integrity. Thus, we suggest that Gab1 is an endogenous enhancer of mTORCs.

Translocation of mTORCs is important for their activation after growth factor stimulation. In this study, we presented evidence to suggest that mTORCs might translocate to the plasma membrane via Gab1-PIP3 binding after EGF stimulation. A recent study indicated that the WD40 domain in raptor binds the lipid PI(3,5)P2, suggesting that mTORC1 translocates to the plasma membrane [35]. However, it has previously been shown that mTORC1 is activated directly by GTP-bound Rheb on the surface of lysosomes [36]. PI(3,5)P2 is generated in lysosomes and at the plasma membrane after stimulation with amino acids and growth factors, and PI(3,5)P2 binding could also contribute to lysosome translocation. Another mechanism of mTORC1 membrane translocation occurs via RalB. Martin et al. (2014) revealed that RalB regulates serum-induced mTORC1 plasma membrane translocation and the activity of mTOR1 in HEK293T cells [27]. Regarding mTORC2, it has been reported that both mTORC2 and AKT were isolated from lipid rafts, which traditionally associate with the plasma membrane [37]. Furthermore, mTORC1 and mTORC2 colocalized with Rac1 at the plasma membrane after serum treatment, and deletion of Rac1 prevented membrane localization of mTORCs [16]. However, knowledge regarding the membrane translocation of mTORCs is minimal.

**Figure 6:**
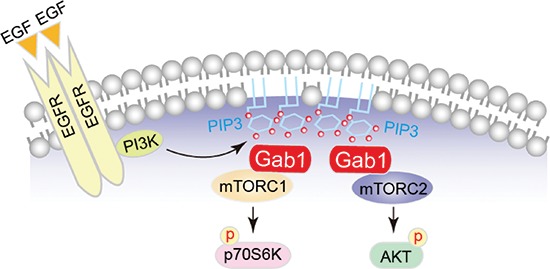
The model of regulation of mTORCs via Gab1 after EGF stimulation in urothelial carcinoma The proposed model that depicts Gab1 regulates mTORC activities via associating with mTORCs and plasma membrane translocation after EGF stimulation in urothelial carcinoma.

Based on *in vivo* and *in vitro* studies, we have identified increased expression of Gab1 and phosphorylation of mTORCs in rats with urothelial carcinoma and human urothelial carcinoma tumor tissues. Taken together, our results highlight the unique role of Gab1 in the regulation of mTORCs after EGF stimulation (Figure 6) as well as the crucial role of Gab1 in urothelial carcinoma. We believe that Gab1 may be a biomarker to predict the diagnosis and drugs response of urothelial carcinoma.

## MATERIALS AND METHODS

### Cell lines, antibodies and reagents

All human urothelial cell carcinoma cell lines were obtained from the American Type Culture Collection (Rockville, MD). The RT4 and T24 cells were cultured in McCoy's 5A medium (Sigma-Aldrich, St. Louis, MO). The E6 and TCCSUP were cultured in DMEM medium (GIBCO^®^ Grand Island), NY. The TSGH8301 and J82 were cultured in RPMI-1640 medium (GIBCO^®^ Grand Island). The grade of these bladder cell line and urothelial carcinoma cell lines: E6 (Normal), RT4 (Grade 1), TSGH8301 (Grade 2) and J82, T24 (Grade 3), respectively [38–41]. All media were supplemented with 10% fetal bovine serum, 100 U/ml penicillin-G, 100 g/ml streptomycin, and 2 mM L-glutamine (CCS; HyClone, Logan, UT). All cell lines were incubated at 37°C with 5% CO_2_. The primary antibodies used in this study included the following: mTOR-pS2481, mTOR-pS2448, mTOR, p70S6K-pT389, ERK, ERK-p, Gab1, AKT (Cell Signaling, Boston, MA), mSin1 (Millipore, Billerica, MA), p70S6K, AKT-pS473, c-myc (Santa Cruz Biotechnology Inc., Santa Cruz, CA), rictor, raptor and beta-actin (GeneTex, Hsinchu, Taiwan). EGF (100 ng/ml) and insulin (2 μg/ml) were purchased from PEPROTECH, and LY294002 (20 μM) was purchased from GIBCO^®^.

### Mutagenesis and cellular transfections with plasmids and siRNA

pCMV-myc-gab1wt was described previously [42]. The Gab1(ΔPH) mutant was amplified by polymerase chain reaction using pCMV-myc-gab1wt as a template with the forward primer 5′-GAATTCGGGGGTTTAATCCAACAGAAGAAGA TCCT-3′ and the reverse primer 5′-GCGGCCGCTCATTTCACACTCTTCGCTGG-3′. The amplicon was subsequently cloned in frame to the *Eco*RI and *Not*I sites of the pCMV-myc vector (Clontech, Mountain View, CA, USA). For exogenous plasmid DNA transfection, the cells (2 × 10^5^) were seeded in 6-cm dishes before transfection. After 24 h in culture, the cells were seeded in 6-cm dishes and transfected with pCMV-myc-gab1wt (3 μg for T24 cells and 1 μg for E6 cells) and pCMV-myc-gab1(ΔPH) (0.5 μg for T24 cells and 1 μg for E6 cells) using the TransIT^®^-2020 transfection reagent according to the manufacturer's instructions (Mirus^®^, Madison, Wisconsin). For siRNA transfections, the cells (1 × 10^5^) were seeded in 6-cm dishes before transfection. After 24 h in culture, the cells seeded in 6-cm dishes and transfected with 10 nM Gab1 siRNA, 10 nM raptor siRNA or 10 nM rictor siRNA (all siRNAs are pool of 3 target-specific 20–25 nt siRNAs designed to knock down gene expression) (Santa Cruz Biotechnology Inc., Santa Cruz, CA) using the INTERFERin siRNA reagent according to the manufacturer's instructions (Polyplus-transfection, New York, NY).

### Western blotting and immunoprecipitation

For western blotting, the cells and patient samples were lysed in RIPA buffer (Millipore, Billerica, Massachusetts) with Halt^TM^ protease & phosphatase inhibitor (Thermo Scientific, Rockford, IL). For immunoprecipitation, the primary antibodies (Gab1, 1:50 and rictor, 1:50) were incubated with Dynabeads^®^ Protein G (Invitrogen Dynal AS, Oslo, Norway) for 1 h at 25°C, followed by incubation with the cell lysates (500 μg-4 mg) for 2 h at 25°C. Total cell extracts and immunoprecipitated proteins were fractionated by SDS-PAGE followed by immunoblotting. The blots were visualized with SuperSignal West Pico chemiluminescent substrate (Thermo Scientific, Rockford, IL) and detected using X-ray film (Fuji Photo Film, Tokyo). The quantitation of the western blot was quantified by Multi Gauge V3.0. The experiments were repeated in triplicate.

### Morphometric analysis and immunofluorescence stain

After fixing bladder tissue in 10% formalin, the lumen was inspected for grossly visible lesions. All immunohistochemical studies were performed on paraffin-embedded sections. The 4-μm-thick deparaffinized sections were incubated with the primary antibodies Gab1 (1:250), AKT-pS473 (1:250) (Santa Cruz Biotechnology Inc., Santa Cruz, CA) and mTOR-pS2481 (1:250) (Abcam, Cambridge, MA). As a negative control, the primary antibody was replaced with normal rabbit IgG, and staining was not performed. All slides were scanned with a Leica DM750. For the immunofluorescence staining, the cells were fixed in 4% paraformaldehyde for 30 min and incubated in 1% Triton-100 for 30 min at 25°C. The primary antibodies Gab1 (1:100) (Santa Cruz Biotechnology Inc., Santa Cruz, CA), rictor (1:1000) and mTOR (1:500) (GeneTex, Hsinchu, Taiwan) were incubated overnight at 4°C. The secondary antibodies Alexa Fluor^®^ 488 goat anti-mouse, goat anti-rabbit; Alexa Fluor^®^ 546 goat anti-mouse, goat anti-rabbit; and Alexa Fluor^®^ 647 goat anti-rabbit (Invitrogen, Grand Island, NY) were incubated for 1 h at 25°C, and the coverslips were mounted in DAPI Fluoromount-G (Southern Biotech, Birmingham, Alabama) and scanned by laser-scanning confocal microscopy (OLYMPUS FLUOVIEW FV1000). The experiments were repeated in triplicate.

### Animal model

The rat model of urothelial carcinoma was induced by adding 0.05% N-butyl-N-(4-hydroxybutyl) nitrosamine (BBN) to the drinking water for 20 weeks. All rats (Control group, 10 rats and BBN group, 10 tars) were sacrificed under pentobarbital anesthesia after 20 weeks of treatment. The protocol was approved by the Animal Care and Research Committee of Taichung Veterans General Hospital (permit number: La-96409).

### Human tumor specimens

All urothelial carcinoma samples and normal bladder tissue from patients were obtained from the Department of Urology at the Taichung Veterans General Hospital, Taichung, Taiwan, with the approval of the Institutional Review Board (IRB number: CG11044).

### Statistics

All data are expressed as the mean ± standard deviation. All statistical analyses were performed using the Statistical Program of Social Sciences software (SPSS version 13.0 for Windows, SPSS Inc., Chicago, IL). Student's *t*-test was used to determine whether there was a significant difference between two means. Statistical significance was defined as a *P* value less than 0.05.

## SUPPLEMENTARY FIGURES



## References

[1] Wu MJ, Lian JD, Yang CR, Cheng CH, Chen CH, Lee WC, Shu KH, Tang MJ (2004). High cumulative incidence of urinary tract transitional cell carcinoma after kidney transplantation in Taiwan. Am J Kidney Dis.

[2] Wu MJ, Chang CH, Chiu YT, Wen MC, Shu KH, Li JR, Chiu KY, Chen YT (2012). Rictor-dependent AKT activation and inhibition of urothelial carcinoma by rapamycin. Urol Oncol.

[3] Wullschleger S, Loewith R, Hall MN (2006). TOR signaling in growth and metabolism. Cell.

[4] Loewith R, Jacinto E, Wullschleger S, Lorberg A, Crespo JL, Bonenfant D, Oppliger W, Jenoe P, Hall MN (2002). Two TOR complexes, only one of which is rapamycin sensitive, have distinct roles in cell growth control. Mol Cell.

[5] Laplante M, Sabatini David M (2012). mTOR Signaling in Growth Control and Disease. Cell.

[6] Yang H, Rudge DG, Koos JD, Vaidialingam B, Yang HJ, Pavletich NP (2013). mTOR kinase structure, mechanism and regulation. Nature.

[7] Sarbassov DD, Ali SM, Kim DH, Guertin DA, Latek RR, Erdjument-Bromage H, Tempst P, Sabatini DM (2004). Rictor, a novel binding partner of mTOR, defines a rapamycin-insensitive and raptor-independent pathway that regulates the cytoskeleton. Curr Biol.

[8] Jacinto E, Facchinetti V, Liu D, Soto N, Wei S, Jung SY, Huang Q, Qin J, Su B (2006). SIN1/MIP1 maintains rictor-mTOR complex integrity and regulates Akt phosphorylation and substrate specificity. Cell.

[9] Sarbassov DD, Guertin DA, Ali SM, Sabatini DM (2005). Phosphorylation and regulation of Akt/PKB by the rictor-mTOR complex. Science.

[10] Wang X, Proud CG (2010). mTORC1 signaling: what we still don't know. J Mol Cell Biol.

[11] Gan X, Wang J, Su B, Wu D (2011). Evidence for Direct Activation of mTORC2 Kinase Activity by Phosphatidylinositol 3,5-Trisphosphate. J Biol Chem.

[12] Zinzalla V, Stracka D, Oppliger W, Hall Michael N (2011). Activation of mTORC2 by Association with the Ribosome. Cell.

[13] Julien LA, Carriere A, Moreau J, Roux PP (2009). mTORC1-Activated S6K1 Phosphorylates Rictor on Threonine 1135 and Regulates mTORC2 Signaling. Mol Cell Biol.

[14] Chen CH, Shaikenov T, Peterson TR, Aimbetov R, Bissenbaev AK, Lee SW, Wu J, Lin HK, Sarbassov DD (2011). ER Stress Inhibits mTORC2 and Akt Signaling Through GSK-3-Mediated Phosphorylation of Rictor. Sci Signal.

[15] Liu P, Gan W, Inuzuka H, Lazorchak AS, Gao D, Arojo O, Liu D, Wan L, Zhai B, Yu Y, Yuan M, Kim BM, Shaik S, Menon S, Gygi SP, Lee TH (2013). Sin1 phosphorylation impairs mTORC2 complex integrity and inhibits downstream Akt signalling to suppress tumorigenesis. Nat Cell Biol.

[16] Saci A, Cantley Lewis C, Carpenter Christopher L (2011). Rac1 Regulates the Activity of mTORC1 and mTORC2 and Controls Cellular Size. Mol Cell.

[17] Huang J, Dibble CC, Matsuzaki M, Manning BD (2008). The TSC1-TSC2 Complex Is Required for Proper Activation of mTOR Complex 2. Mol Cell Biol.

[18] Cao C, Huang X, Han Y, Wan Y, Birnbaumer L, Feng GS, Marshall J, Jiang M, Chu WM (2009). Galpha(i1) and Galpha(i3) are required for epidermal growth factor-mediated activation of the Akt-mTORC1 pathway. Sci Signal.

[19] Holgado-Madruga M, Emlet DR, Moscatello DK, Godwin AK, Wong AJ (1996). A Grb2-associated docking protein in EGF- and insulin-receptor signalling. Nature.

[20] Lehr S, Kotzka J, Herkner A, Klein E, Siethoff C, Knebel B, Noelle V, Bruning JC, Klein HW, Meyer HE, Krone W, Muller-Wieland D (1999). Identification of tyrosine phosphorylation sites in human Gab-1 protein by EGF receptor kinase *in vitro*. Biochemistry.

[21] Gu H, Neel BG (2003). The ‘Gab’ in signal transduction. Trends in Cell Biol.

[22] Lemmon MA, Falasca M, Schlessinger J, Ferguson K (1997). Regulatory recruitment of signalling molecules to the cell membrane by pleckstrinhomology domains. Trends Cell Biol.

[23] Rodrigues GA, Falasca M, Zhang Z, Ong SH, Schlessinger J (2000). A novel positive feedback loop mediated by the docking protein Gab1 and phosphatidylinositol 3-kinase in epidermal growth factor receptor signaling. Mol Cell Biol.

[24] Maroun CR, Holgado-Madruga M, Royal I, Naujokas MA, Fournier TM, Wong AJ, Park M (1999). The Gab1 PH domain is required for localization of Gab1 at sites of cell-cell contact and epithelial morphogenesis downstream from the met receptor tyrosine kinase. Mol Cell Biol.

[25] Isakoff SJ, Cardozo T, Andreev J, Li Z, Ferguson KM, Abagyan R, Lemmon MA, Aronheim A, Skolnik EY (1998). Identification and analysis of PH domain-containing targets of phosphatidylinositol 3-kinase using a novel *in vivo* assay in yeast. EMBO J.

[26] Colquhoun AJ, Mellon JK (2002). Epidermal growth factor receptor and bladder cancer. Postgrad Med J.

[27] Martin Timothy D, Chen X-W, Kaplan Rebecca EW, Saltiel Alan R, Walker Cheryl L, Reiner David J, Der Channing J (2014). Ral and Rheb GTPase Activating Proteins Integrate mTOR and GTPase Signaling in Aging, Autophagy, and Tumor Cell Invasion. Mol Cell.

[28] Ching CB, Hansel DE (2010). Expanding therapeutic targets in bladder cancer: the PI3K/Akt/mTOR pathway. Lab Invest.

[29] White NM, Masui O, Newsted D, Scorilas A, Romaschin AD, Bjarnason GA, Siu KW, Yousef GM (2014). Galectin-1 has potential prognostic significance and is implicated in clear cell renal cell carcinoma progression through the HIF/mTOR signaling axis. Br J Cancer.

[30] Cornu M, Albert V, Hall MN (2013). mTOR in aging, metabolism, and cancer. Curr Opin Genet Dev.

[31] Milella M, Felici A (2011). Biology of metastatic renal cell carcinoma. J Cancer.

[32] Pearce LR, Komander D, Alessi DR (2010). The nuts and bolts of AGC protein kinases. Nat Rev Mol Cell Biol.

[33] Peterson TR, Laplante M, Thoreen CC, Sancak Y, Kang SA, Kuehl WM, Gray NS, Sabatini DM (2009). DEPTOR is an mTOR inhibitor frequently overexpressed in multiple myeloma cells and required for their survival. Cell.

[34] Khanna N, Fang Y, Yoon MS, Chen J (2013). XPLN is an endogenous inhibitor of mTORC2. Proc Natl Acad Sci U S A.

[35] Bridges D, Ma JT, Park S, Inoki K, Weisman LS, Saltiel AR (2012). Phosphatidylinositol 3,5-bisphosphate plays a role in the activation and subcellular localization of mechanistic target of rapamycin 1. Mol Biol Cell.

[36] Saito K, Araki Y, Kontani K, Nishina H, Katada T (2005). Novel role of the small GTPase Rheb: its implication in endocytic pathway independent of the activation of mammalian target of rapamycin. J Biochem.

[37] Partovian C, Ju R, Zhuang ZW, Martin KA, Simons M (2008). Syndecan-4 regulates subcellular localization of mTOR Complex2 and Akt activation in a PKCalpha-dependent manner in endothelial cells. Mol Cell.

[38] Cheng HL, Trink B, Tzai TS, Liu HS, Chan SH, Ho CL, Sidransky D, Chow NH (2002). Overexpression of c-met as a prognostic indicator for transitional cell carcinoma of the urinary bladder: a comparison with p53 nuclear accumulation. J Clin Oncol.

[39] Masters JR, Hepburn PJ, Walker L, Highman WJ, Trejdosiewicz LK, Povey S, Parkar M, Hill BT, Riddle PR, Franks LM (1986). Tissue culture model of transitional cell carcinoma: characterization of twenty-two human urothelial cell lines. Cancer Res.

[40] Shen CH, Shee JJ, Wu JY, Lin YW, Wu JD, Liu YW (2010). Combretastatin A-4 inhibits cell growth and metastasis in bladder cancer cells and retards tumour growth in a murine orthotopic bladder tumour model. Br J Pharmacol.

[41] Yeh MY, Yu DS, Chen SC, Lin MS, Chang SY, Ma CP, Han SH (1988). Establishment and characterization of a human urinary bladder carcinoma cell line (TSGH-8301). J Surg Oncol.

[42] Chan PC, Sudhakar JN, Lai CC, Chen HC (2009). Differential phosphorylation of the docking protein Gab1 by c-Src and the hepatocyte growth factor receptor regulates different aspects of cell functions. Oncogene.

