# Supplementary material for: Gab1 is essential for membrane translocation, activity and integrity of mTORCs after EGF stimulation in urothelial cell carcinoma
Source: Oncotarget. 2015 Jan 20;6(3):1478–89. doi: 10.18632/oncotarget.2756 (PMC4359308; doi:10.18632/oncotarget.2756)
Supplement: Supplementary file 1 [file oncotarget-06-1478-s001.pdf]

SUPPLEMENTARY FIGURES

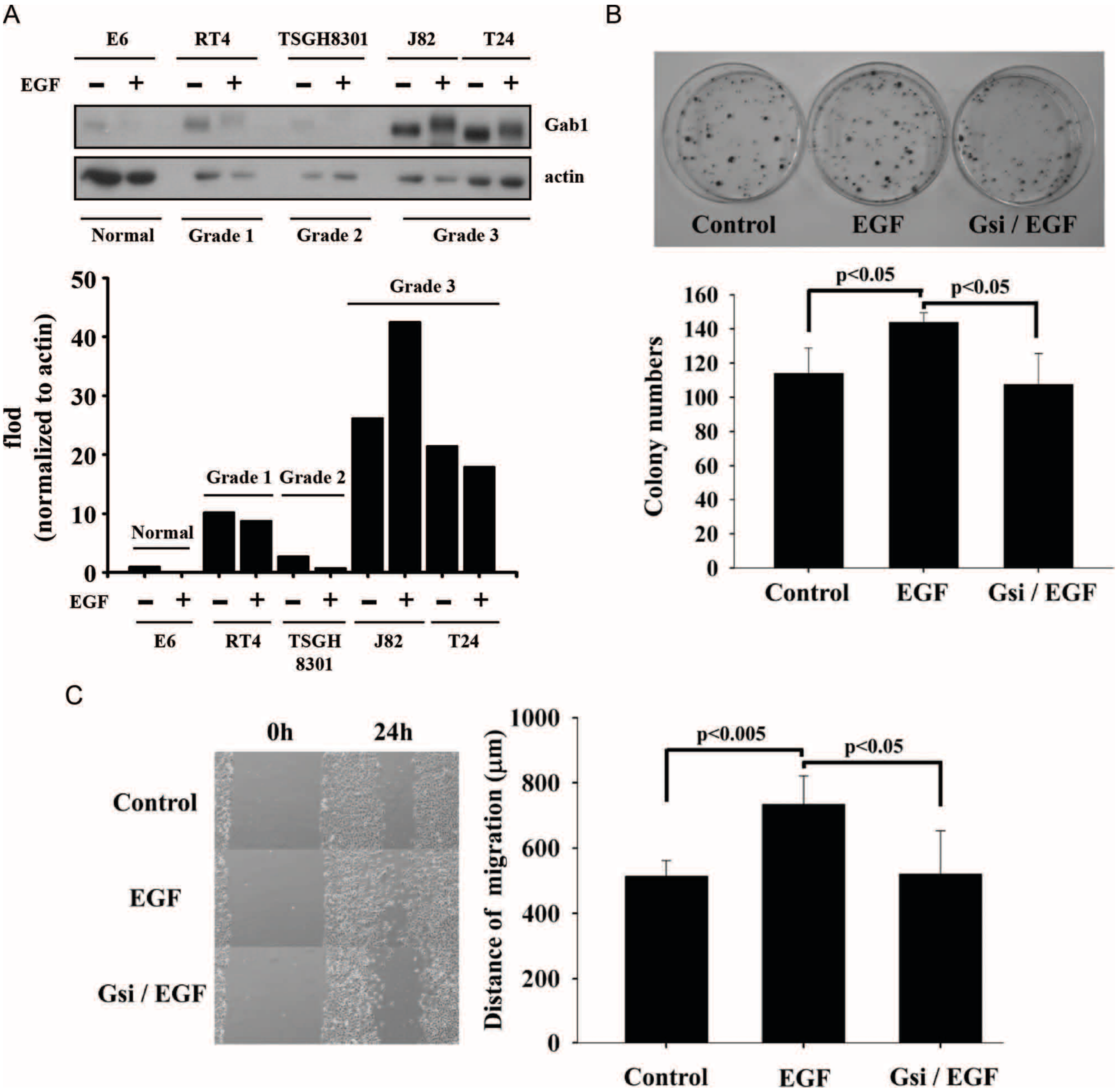

Supplementary Figure S1: The supplementary data for Figure 1. **(A)** The expression level of Gab1 was found to be weaker in normal urothelial cell lines (E6, normal) and low-grade urothelial carcinoma cell lines (RT4, Grade I; TSGH8301, Grade II) and higher in high-grade urothelial carcinoma cell line (J82, T24, Grade III). **(B)** Knockdown of Gab1 reduced colony formation numbers of T24 cells upon EGF stimulation. Gsi: Gab1 siRNA. **(C)** Knockdown of Gab1 inhibited cell migration ability of T24 cells upon EGF stimulation. Gsi: Gab1 siRNA.

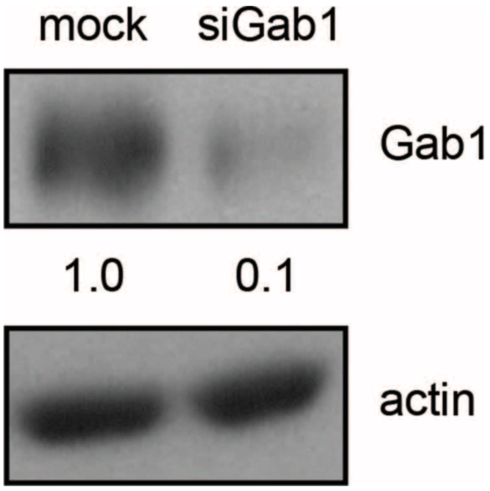

Supplementary Figure S2: Knockdown efficiency of Gab1 for Figure 2.

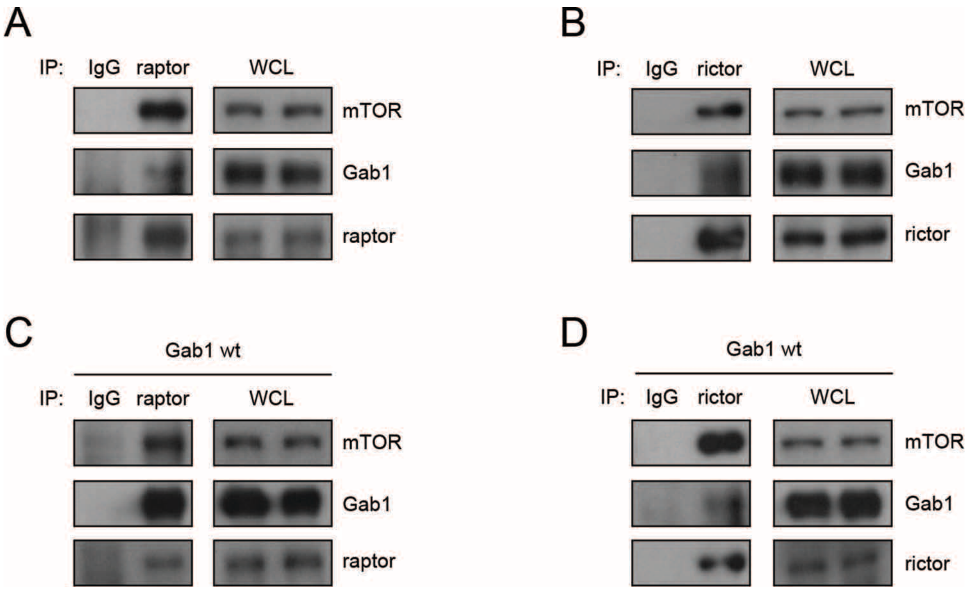

Supplementary Figure S3: The negative control of co-immunoprecipitation for Figure 5.
